# Oxidized LDL Receptor 1 (*OLR1*) as a Possible Link between Obesity, Dyslipidemia and Cancer

**DOI:** 10.1371/journal.pone.0020277

**Published:** 2011-05-26

**Authors:** Magomed Khaidakov, Sona Mitra, Bum-Yong Kang, Xianwei Wang, Susan Kadlubar, Giuseppe Novelli, Vinay Raj, Maria Winters, Weleetka C. Carter, Jawahar L. Mehta

**Affiliations:** 1 Department of Internal Medicine, College of Medicine, and the Central Arkansas Veterans Healthcare System, Little Rock, Arkansas, United States of America; 2 Division of Medical Genetics, College of Medicine, University of Arkansas for Medical Sciences, Little Rock, Arkansas, United States of America; 3 Emory University, Atlanta, Georgia, United States of America; 4 Department of Genetics, University of Rome “Tor Vergata”, Rome, Italy; Istituto Dermopatico dell'Immacolata, Italy

## Abstract

Recent studies have linked expression of lectin-like ox-LDL receptor 1 (*OLR1*) to tumorigenesis. We analyzed microarray data from *Olr1* knockout (KO) and wild type (WT) mice for genes involved in cellular transformation and evaluated effects of *OLR1* over-expression in normal mammary epithelial cells (MCF10A) and breast cancer cells (HCC1143) in terms of gene expression, migration, adhesion and transendothelial migration. Twenty-six out of 238 genes were inhibited in tissues of OLR1 KO mice; the vast majority of OLR1 sensitive genes contained NF-κB binding sites in their promoters. Further studies revealed broad inhibition of NF-kB target genes outside of the transformation-associated gene pool, with enrichment themes of defense response, immune response, apoptosis, proliferation, and wound healing. Transcriptome of *Olr1* KO mice also revealed inhibition of *de novo* lipogenesis, rate-limiting enzymes fatty acid synthase (*Fasn*), stearoyl-CoA desaturase (*Scd1*) and ELOVL family member 6 (*Elovl6*), as well as lipolytic phospholipase A2 group IVB (*Pla2g4b*). In studies comparing MCF10A and HCC1143, the latter displayed 60% higher *OLR1* expression. Forced over-expression of *OLR1* resulted in upregulation of NF-κB (p65) and its target pro-oncogenes involved in inhibition of apoptosis (*BCL2*, *BCL2A1*, *TNFAIP3*) and regulation of cell cycle (*CCND2*) in both cell lines. Basal expression of *FASN*, *SCD1* and *PLA2G4B*, as well as lipogenesis transcription factors *PPARA*, *SREBF2* and *CREM*, was higher in HCC1143 cells. Over-expression of *OLR1* in HCC1143 cells also enhanced cell migration, without affecting their adherence to TNFα-activated endothelium or transendothelial migration. On the other hand, *OLR1* neutralizing antibody inhibited both adhesion and transmigration of untreated HCC1143 cells. We conclude that *OLR1* may act as an oncogene by activation of NF-kB target genes responsible for proliferation, migration and inhibition of apoptosis and *de novo* lipogenesis genes.

## Introduction

*OLR1*, a lectin-like scavenger receptor, is highly conserved in mammals [1] and it is capable of recognizing several ligands including the protein moiety of oxidized-LDL (ox-LDL), advanced glycation end-products, gram-positive and gram-negative bacteria and apoptotic cells [2]. *OLR1* is primarily expressed in vascular cells and vasculature-rich organs [3], and its activation by a wide range of stimuli indicative of dyslipidemia, inflammation and damage initiates several signaling cascades including MAPKs, other protein kinases as well as transcription factors NF-κB and AP-1 [4], [5].

Overexpression of *OLR1* has been shown in cellular components of atherosclerotic lesions [6]. Deletion of *Olr1* in *Ldlr* knockout (KO) mice results in much smaller atherosclerotic lesions associated with a drastic reduction of inflammation in the aortic wall [7]. *Olr1* abrogation also attenuates angiotensin II-induced hypertension [8]. Similarly, abrogation of *Olr1* reduces the extent of ischemia/reperfusion injury [9].

An association between obesity and atherosclerotic disease states in humans is well established [10], [11]. Associations with obesity have been found for various cancers, including breast and prostate neoplasms [12], [13], suggesting a mechanistic overlap in the pathobiology of atherogenesis and tumorigenesis. Recently, *Olr1*, acting through NF-κB mediated inflammatory signaling, was strongly implicated in carcinogenesis [14].

The focus of the present study was to further elucidate role of *OLR1* as an oncogene based on the premise that as a sensor of dyslipidemia and a molecule involved in NF-kB activation, *OLR1* may be a link between dyslipidemia and cancer. The first part of the study was based on microarray analysis of wild-type (WT) and *olr1* KO mice. The second part defines the relationship between *olr1* and apoptosis and lipogenesis genes in breast cancer cell line HCC1143 and migration and adhesion of these cells.

## Results

### Comparison of *OLR1* KO and transformation transcriptomes

**Figure 1:**
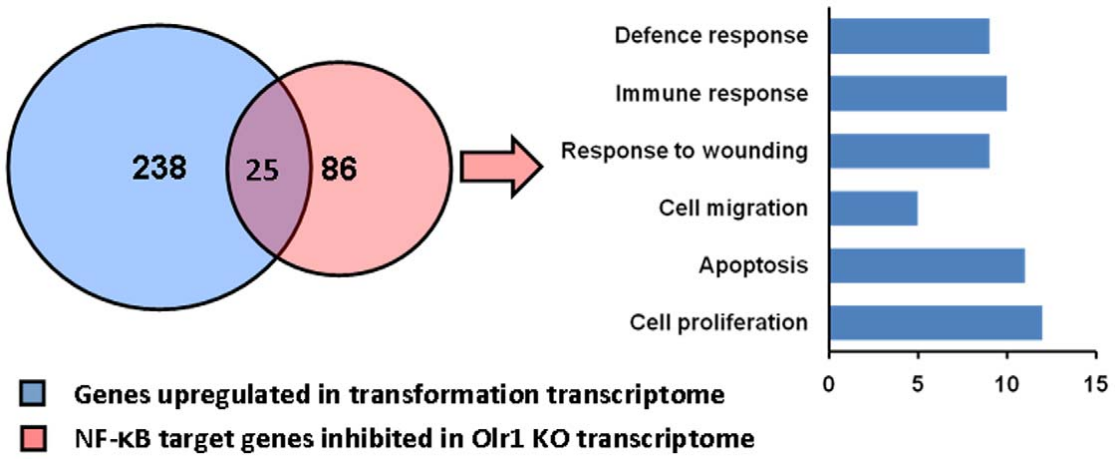
*Olr1* deletion results in broad inhibition of NF-κB target genes. A diagram depicting a set of overlapping genes between transformation and *Olr1* KO transcriptomes. From the set of 238 genes upregulated during transformation, 26 genes were found to be inhibited in *Olr1* KO mice. Vast majority of these genes carried NF-κB sites in their proximal promoter sequences. In total, 86 NF-κB target genes were found to be inhibited in *Olr1* KO mice with enrichment for regulation of apoptosis (p = 0.0002), proliferation (p = 0.00003), wound healing (p = 0.0002), defense response (p = 0.0011), immune response (p = 0.0003) and cell migration (p = 0.0009).

The principal findings from the analysis of microarray data from the hearts of wild-type (WT) and *Olr1* KO from our group have been reported elsewhere [15]. An additional analysis against the overlapping set of genes (n = 238) found to be upregulated during transformation of two isogenic cell types [14] revealed that 26 genes from this list were inhibited in the *Olr1* KO transcriptome by at least 20% (Table 1). Among these were various components of immune response (*Isg20*, *C1s*, *S1r*, *Ifrd1*) and a number of transcription factors including well-known oncogenes (*JunB*, *Rel*, *Irf2*, *Crem*). Promoter analysis of resulting set of genes [16] identified their enrichment for NF-κB binding sites (p<0.03) which were located within 500 nucleotides proximal to transcriptional start sites in all but one (*C1s*) *Olr1* sensitive genes (n = 25, Fig. 1).

**Table 1:** A list of transformation related genes influenced by *Olr1* deletion in mice.

Gene	REFSEQ ID	Symbol	Fold change	P value
***Inhibited***				
interferon-stimulated protein	NM_020583	*Isg20*	−3.26	0.007
matrix metallopeptidase 3	NM_0011135271	*Mmp3*	−2.05	0.014
reticuloendotheliosis oncogene	NM_009044	*Rel*	−2.02	0.011
complement component 1, s subcomponent	NM_144938	*C1s*	−1.76	0.008
complement component 1, r subcomponent	NM_023143,	*C1r*	−1.61	0.001
suppressor of cytokine signaling 3	NM_007707	*Socs3*	−1.57	0.003
Jun-B oncogene	NM_008416	*Junb*	−1.56	0.0009
interferon regulatory factor 2	NM_008391	*Irf2*	−1.51	0.044
fibroblast activation protein	NM_007986	*Fap*	−1.44	0.005
synaptosomal-associated protein 23	NM_009222	*Snap23*	−1.41	0.033
N-myc (and STAT) interactor	NM_001141949	*Nmi*	−1.41	0.0019
cAMP responsive element modulator	NM_013498	*Crem*	−1.40	0.043
pentraxin related gene	NM_008987	*Ptx3*	−1.38	0.052
MAPK kinase kinase 7 interacting protein 2	NM_138667	*Map3k7ip2*	−1.37	0.041
TNF receptor superfam. member 21	NM_178589	*Tnfrsf21*	−1.35	0.011
Acetylglucosamine (GlcNAc) transferase	NM_139144	*Ogt*	−1.35	0.002
Ras-GTPase-activating protein SH3-domain binding protein 1	NM_013716	*G3bp1*	−1.35	0.003
Rho family GTPase 3	NM_028810	*Rnd3*	−1.31	0.013
interferon-related developmental regulator 1	NM_013562	*Ifrd1*	−1.29	0.026
oxidation resistance 1	NM_130885	*Oxr1*	−1.29	0.003
solute carrier family 2 member 3	NM_011401	*Slc2a3*	−1.28	0.044
annexin A7	NM_009674	*Anxa7*	−1.28	0.001
karyopherin (importin) alpha 2	NM_010655	*Kpna2*	−1.27	0.014
prostaglandin-endoperoxide synthase 1	NM_008969	*Ptgs1*	−1.26	0.031
lectin, galactose binding, soluble 8	NM_018886	*Lgals8*	−1.20	0.014
***Stimulated***				
ser (or cys) peptidase inhibitor, clade. E, memb 2	NM_009255	*Serpine2*	1.26	0.018
succinate dehydrogenase complex, subunit C	NM_025321	*Sdhc*	1.29	0.017
E26 avian leukemia oncogene 2, 3′ domain	NM_011809	*Ets2*	1.35	0.001
NF-κ light polypeptide gene enhancer in B-cells inhibitor, alpha	NM_010907	*Nfkbia*	1.36	0.002
GTP binding protein 2	NM_001145979	*Gtpbp2*	1.41	0.008
mitogen-activated protein kinase kinase kinase 5	NM_008580	*Map3k5*	1.51	0.005
SRY-box containing gene 4	NM_009238	*Sox4*	1.54	0.007

### *OLR1* deletion results in a broad inhibition of NF-κB target genes

Further search for NF-κB regulated genes using a list compiled from available web based databases and the literature revealed that inhibition of the p65 subunit observed in the *Olr1* KO transcriptome was complemented with upregulation of inhibitory *Ikbα* subunit (1.36 fold, p = 0.002, Table 1) and accompanied by significant downregulation of several (n = 61) NF-κB target genes (Table 2). The combined set of *Olr1* sensitive NF-κB target genes displayed enrichment for regulation of apoptosis (p = 0.0002), proliferation (p = 0.00003), wound healing (p = 0.0002), defense response (p = 0.0011), immune response (p = 0.0003) and cell migration (p = 0.0009) (Figure 1). Among the genes involved in apoptosis (n = 11) and cellular proliferation (n = 12), 6 and 5, respectively, were negative regulators (David Bioinformatics Database, 17).

**Table 2:** NF-κB target genes outside of transformation pool significantly inhibited in Olr1 knockout mice (more than 1.2-fold).

Gene Title	Gene Symbol^a^	Fold change	P value	A	W	P	D	M
colony stimulating factor 2	*Csf2^1,2^*	-3.55	0.0148	•		•		
phospholipase A2, group IVB (cytosolic)	*Pla2g4b^1^*	-3.29	0.0079					
lectin, galactose binding, soluble 3	*Lgals3^1^*	-2.93	0.0302					
BCL 2 related protein A1a	*Bcl2a1^1,2,3^*	-2.90	0.0304	•				
chitinase 3-like 1	*Chi3l1^2^*	-2.80	0.0145					
lymphotoxin A	*Lta^1^*	-2.73	0.0007	•	•	•	•	
hepcidin antimicrobial peptide	*Hamp^2^*	-2.50	0.0518				•	
immunoglobulin heavy chain complex	*Igh^2^*	-2.34	0.0006					
glucosaminyl (N-acetyl) transferase 1, core 2	*Gcnt1^2^*	-2.23	0.0216					
fos-like antigen 2	*Fosl2^2^*	-2.22	0.0012			•		
NFκB inhibitor, ε	*Nfkbie^2^*	-2.20	0.0321					
selectin, platelet	*Selp^1^*	-2.20	0.0309		•		•	•
complement factor B	*Cfb^1^*	-2.15	0.0079		•		•	
deiodinase, iodothyronine, type II	*Dio2^2^*	-2.07	0.0039					
FBJ osteosarcoma oncogene	*Fos^2^*	-2.03	0.0270					
interleukin 17A	*Il17a^2^*	-2.00	0.0103		•		•	
thrombospondin 1	*Thbs^2^*	-1.94	0.0146		•		•	•
CD3 antigen, gamma polypeptide	*Cd3g^1^*	-1.92	0.0020	•				
LPS-induced TN factor	*Litaf^3^*	-1.91	0.0072					
CD48 antigen	*Cd48^1^*	-1.77	0.0228					
prostaglandin E synthase	*Ptges^2^*	-1.61	0.0484			•		
chemokine (C-X-C motif) ligand 5	*Cxcl5^1^*	-1.60	0.0055		•		•	
HSP90, α (cytosolic), class A member 1	*Hsp90aa1^2^*	-1.59	0.0355					
CD209f antigen	*Cd209f^2^*	-1.56	0.0067					
vascular cell adhesion molecule 1	*Vcam1^1,2^*	-1.54	0.0007					
coagulation factor III	*F3^1^*	-1.54	0.0111		•			
twist gene homolog 1 (Drosophila)	*Twist1^2^*	-1.52	0.0092					
B-cell leukemia/lymphoma 2	*Bcl2^1,2,3^*	-1.52	0.0201	•	•	•	•	•
cyclin D2	*Ccnd2^3^*	-1.51	0.0161			•		
dihydropyrimidine dehydrogenase	*Dpyd^2^*	-1.48	0.0062					
CASP8 and FADD-like apopt. regulator	*Cflar^1,2,3^*	-1.44	0.0544	•				
related RAS viral oncogene homolog 2	*Rras2^3^*	-1.43	0.0476					•
CD274 antigen	*Cd274^2^*	-1.42	0.0273			•		
immediate early response 3	*Ier3^1,2,3^*	-1.41	0.0250					
Fas (TNFRSF6)-assoc. via death domain	*Fadd^3^*	-1.39	0.0272	•				
TNF, alpha-induced protein 3	*Tnfaip3^1,2,3^*	-1.38	0.0520					
Kruppel-like factor 10	*Klf10^3^*	-1.38	0.0267	•		•		
NUAK family, SNF1-like kinase, 2	*Nuak2^2^*	-1.37	0.0300	•				
TNF receptor superfamily, member 21	*Tnfrsf21^1^*	-1.35	0.0113					
interferon regulatory factor 7	*Irf7^1,2^*	-1.35	0.0173					
transglutaminase 2, C polypeptide	*Tgm2^1^*	-1.31	0.0390	•		•		
CD82 antigen	*Cd82^3^*	-1.31	0.0308					
phosphodiesterase 7A	*Pde7a^2^*	-1.31	0.0123					
heparanase	*Hpse^2^*	-1.29	0.0390					
ATP-binding cass., subf. B, memb. 1A	*Abcb1a^1^*	-1.28	0.0298					
midkine	*Mdk^2^*	-1.27	0.0502		•			
MAD homolog 7 (Drosophila)	*Smad7^3^*	-1.27	0.0195					
solute carrier family 3, member 2	*Slc3a2^2^*	-1.27	0.0355					
Proteasome subunit, beta type 9	*Psmb9^1,2^*	-1.25	0.0264					
cyclin D binding myb-like TF 1	*Dmtf1^2^*	-1.25	0.0224					
beta-2 microglobulin	*B2m^1,2^*	-1.24	0.0007				•	
adenosine A1 receptor	*Adora1^1^*	-1.23	0.0215	•		•		•
nuclear receptor subf. 3, gr. C, member 1	*Nr3c1^2^*	-1.22	0.0540	•				
platelet derived GF, B polypeptide	*Pdgfb^1,2^*	-1.22	0.0152			•		•
cAMP responsive element bind. protein 3	*Creb3^2^*	-1.21	0.0306					

Legend: (^a^)- a list of NFkB target genes was compiled from the following web-based databases: 1 - http://bioinfo.lifl.fr/NF-KB/; 2 -http://people.bu.edu/gilmore/nf-kb/target/index.html#cyto; and 3 - http://www.broadinstitute.org/mpr/publications/projects/Lymphoma/FF_NFKB_suppl_revised.pdf. The genes whose expression was significantly altered by OLR1 deletion was analyzed for enrichment themes using DAVID bioinformatic database. The enriched themes included regulation of apoptosis, “A”; Wound healing,“W”; Cell proliferation, “P”; Defense response,“D”; Cell migration, “M”.

### *OLR1* deletion suppresses lipogenesis genes

**Figure 2:**
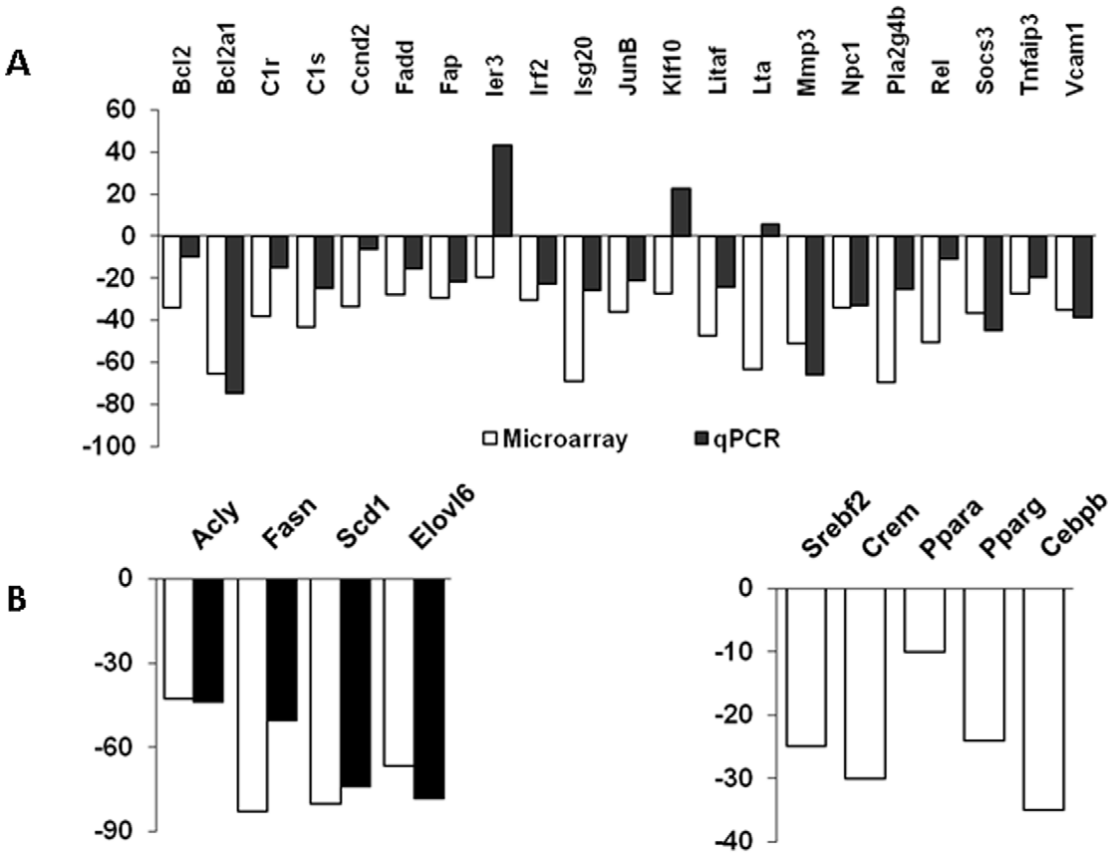
qPCR validation of microarray data. **A.** qPCR validation of select genes from overlapping set (from Table 1); **B.** Expression of lipogenesis genes and transcription factors in *olr1* KO mice. White bars – microarray data; black bars – qPCR data. All P values<0.05.

The microarray findings were validated for select genes using quantitative real-time PCR. For most of the tested genes, the transcriptional shifts in *Olr1* KO observed in microarrays were confirmed (Figure 2A). In addition, *Olr1* deletion resulted in inhibition of key enzymes for lipogenesis (Figure 2B), including ATP citrate lyase (*Acly*), acetyl-Coenzyme A carboxylase alpha (*Acaca*), fatty acid synthase (*Fasn*), stearoyl-CoA Desaturase 1 (*Scd1*) and ELOVL family member 6 (*Elovl6*). It is of interest that none of the *Olr1*-sensitive lipid metabolism related genes carried NF-κB binding sites in their promoters. This suggests that the effects of *Olr1* on lipogenesis may be independent from its NF-κB signaling arm. On the other hand, several lipid metabolism transcription factors, including sterol regulatory element binding factor 2 (*Srebf2*), cAMP responsive element modulator (*Crem*), peroxisome proliferator activated receptors alpha and gamma (*Ppara* and *Pparg*), as well as CCAAT/enhancer binding protein (C/EBP) beta, were found to be downregulated in *Olr1* KO mice (Figure 2B).

### Effects of *OLR1* overexpression in normal epithelial and cancer cells

**Figure 3:**
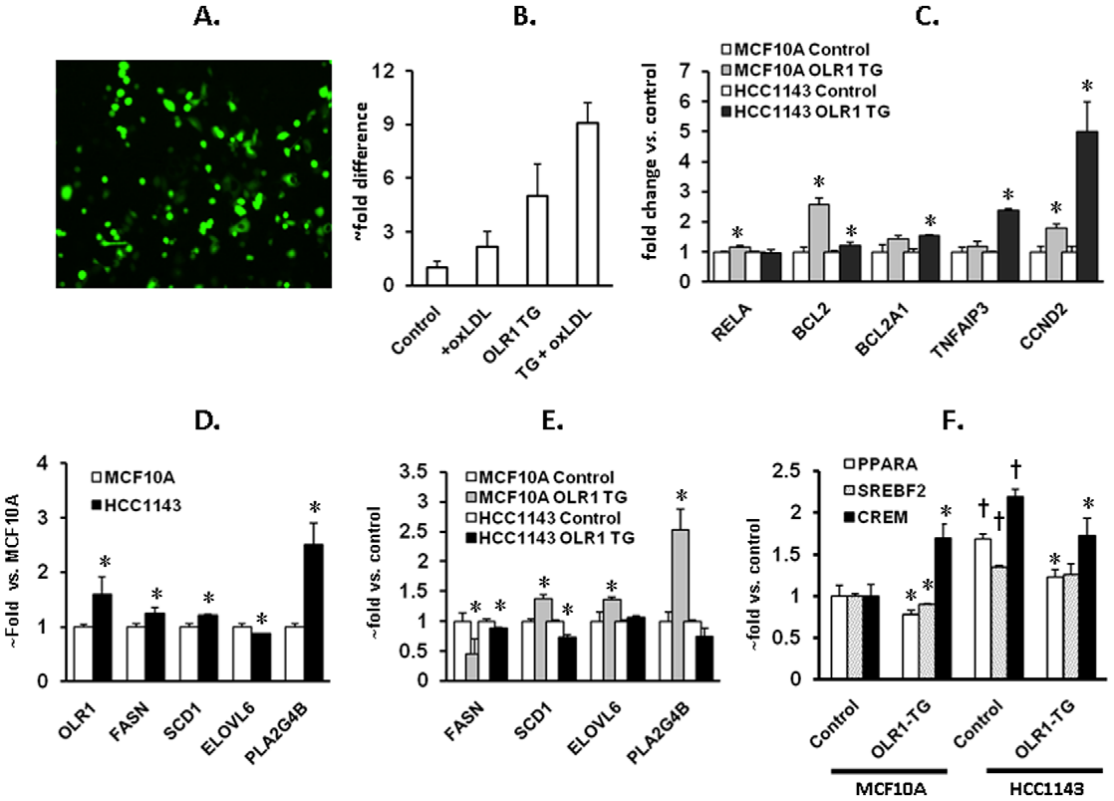
Effects of *OLR1* overexpression on transcription of genes involved in apoptosis, proliferation and lipogenesis in MCF10a and HCC1143 cells. These cells were transfected with either empty vector or *OLR1* cDNA (Origene, Rockville, MD) using Lipofectamine 2000 (Invitrogen). Transfection efficiency (70–80%) was evaluated using GFP vector. RNA was extracted 48 hours post-transfection, converted into cDNA and the expression of genes was determined by quantitative PCR. **A.** Efficiency of transfection (cells transfected with GFP vector). **B.** Quantitative PCR plot. Note the enhancement of OLR1 expression in both control and OLR1-transfected cultures in response to ox-LDL. **C.** Expression of genes involved in apoptosis and proliferation. In order to stimulate *OLR1* associated signaling requiring OLR1-ligand interaction, *OLR1* transfected cells were treated with 40 µg/ml ox-LDL for 24 hours; graphs represent comparison with untreated control cells transfected with empty vector; **D.** Basal expression of *OLR1*, *PLA2G4B* and lipogenesis genes in normal human mammary epithelial cells (MCF10A) and breast cancer cells (HCC1143); **E.** Expression of *OLR1*, *PLA2G4B* and lipogenesis genes in MCF10a and HCC1143 cells transfected with OLR1 treated according to the protocol described above. **F.** Expression of lipogenesis transcription factors in MCF10a and HCC1143 cells transfected with OLR1 and treated according to the protocol described above. All experiments were conducted in triplicates. (*) p<0.05 compared to respective control; (†) – p<0.05 compared to MCF10A.

Transfection of MCF10A and HCC1143 cells with *OLR1* expression vector resulted in 5 to 8-fold increase of *OLR1* expression, which falls within the range OLR1 upregulation. In epithelial cells exposed to ox-LDL [18]. This led to modest upregulation of *RELA* (p65) and significant increases in RNA message for *BCL2, BCL2A1, TNFAIP3 and CCND2* (Figure 3B). Compared to MCF10A cells, HCC1143 cells displayed increased basal levels of *OLR1* (59%, p<0.05), *FASN* (24%, p<0.03), *SCD1* (21%, p<0.01) and *PLA2G4B* (153%, p<0.01) (Figure 3C). The response from lipogenesis genes to *OLR1* transfection varied in these cell lines (Figure 3D). In MCF10A cells, over-expression of *OLR1* significantly stimulated transcription of *SCD1* (37%, p<0.02), *ELOVL6* (38%, p<0.05) and *PLA2G4B* (153%, p<0.02) concomitant with upregulation of *CREM*, whereas in HCC1143 cells *CREM* transcription declined and *SCD1* and *PLA2G4B* were inhibited compared with control cultures transfected with empty vector.

### Over-expression of *OLR1* facilitates wound healing, but has no effect on adhesion

**Figure 4:**
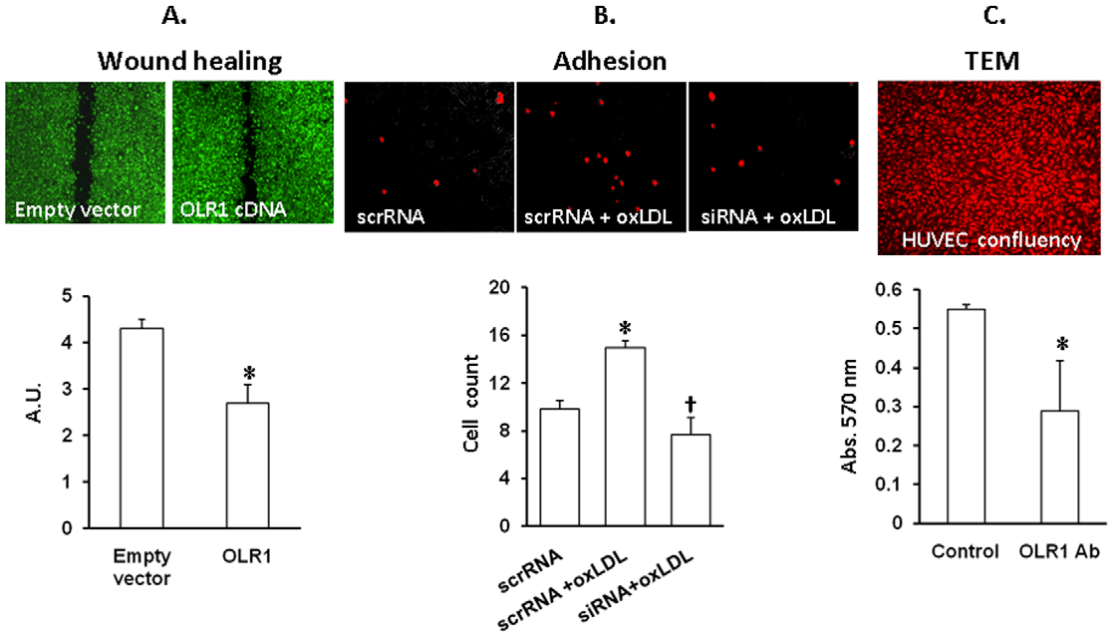
Phenotypic consequences of *OLR1 overexpression or inhibition*. **A.** Wound healing assay. Upper panel - representative images of wound healing assay performed using HCC1143 cells transfected with either empty plasmid or *OLR1* cDNA vector; Lower panel – graph depicting the distance between edges of the wound after 36 hours of incubation. (*) – p<0.01; **B.** Adhesion assay. Upper panel- representative images of adherent non-transfected HCC1143 cells loaded with CellTracker Red CMTX (Invitrogen, Carlbad, CA) and applied to non-activated or activated (50 µg/ml oxLDL, 4 hrs) confluent HUVECs transfected with OLR1 Silencer or scrambled siRNA. Lower panel - graph depicting the number of adherent cells averaged from multiple fields of view in triplicate cultures. (*) – p<0.05 compared to non-activated control (“scrRNA”); (†) - p<0.05 compared to scrambled RNA; **C.** Colorimetric transendothelial migration assay. Upper panel – verification of the confluence of HUVECs on the membranes by staining cells with CellTracker Red CMTX. Lower panel – absorbance values of stain extracted from the cells migrated through TNFα-activated endothelial monolayer in presence of *OLR1* neutralizing antibody or human IgG (Control).

It has been reported that inhibition of *OLR1* using siRNAs resulted in reduction of tumor growth *in vivo* and cancer cell migration *in vitro* [14]. In order to evaluate effects of *OLR1* upregulation, we transfected HCC1143 cells with either empty plasmid or *OLR1* expression vector and tested migration in a wound healing assay (Figure 4A). Over-expression of *OLR1* was confirmed using qPCR. Cells with upregulated *OLR1* bridged the wounds much faster than control cultures (p<0.001). In contrast, adhesion and transendothelial migration of *OLR1* transfected cancer cells did not differ from control values (not shown).

However, basal adhesion and transendothelial migration of non-transfected HCC1143 cells were significantly attenuated when OLR1 was inhibited or neutralized in endothelial cells using siRNA or pre-treating cells with *OLR1* neutralizing antibody (Figure 4BC).

## Discussion

This study suggests multiple potential links between *OLR1* and susceptibility to cancer. First, the microarray database in the mice with *Olr1* abrogation exhibited a marked reduction in expression of NF-kB target genes involved in cellular transformation [14], as well as genes related to lipogenesis. Second, over-expression of *OLR1* in a human cancer cell line showed significant upregulation of several genes with oncogenic properties and a significant increase in cell migration.

Our microarray analysis showed that the vast majority of genes reported to be upregulated during cell transformation (but inhibited in *Olr1* KO mice) carried NF-kB binding sites in their promoters (Table 1). Furthermore, a significant portion of NF-kB target genes outside of the transformation-related pool, especially those involved in regulation of apoptosis, proliferation and migration, were also down-regulated in the *Olr1* KO transcriptome (Table 2). In the mouse *Olr1* KO microarray, both B-cell leukemia/lymphoma 2 related protein A1 (*Bcl2a1*) and *Bcl2* were transcriptionally inhibited. *Bcl2a1* is an upstream negative regulator of the mitochondriocentric mode of apoptosis *via* prevention of cytochrome c release into the cytoplasm, which is required for initiation of the apoptotic cascade. It has been reported that enhanced synthesis of *BCL2A1* and *BCL-XL* are the underlying cause of about 1000-fold greater resistance of subsets of chronic lymphocytic leukemia cells [19]. TNFα-induced protein 3 (*TNFAIP3*) is a key regulator of inflammation and immunity involved in the development of various autoimmune diseases and it also desensitizes cells from TNFα-induced cytotoxicity and was shown to be anti-apoptotic in breast cancer MCF7S1 cells [20]. Inhibition of *TNFAIP3* compromises growth and survival of glioblastoma stem cells *via* inhibition of cell cycle progression and NF-κB activity, and increases survival of mice bearing glioblastome xenografts [21].

We observed a significant reduction of *Ccnd2* message in *Olr1* KO mice and its multi-fold upregulation in *OLR1* transgenic HCC1143 cells. Cyclin D2 is a highly conserved regulator of cyclin-dependent kinases 4 and 6 responsible for of G1/S transition. This gene is epigenetically silenced in the majority of breast cancers [22]. Its overexpression in LNCaP cells results in an impediment to proliferation and increased apoptosis [23]. In addition, *Olr1* deletion appeared to compromise the entire technological chain of *de novo* lipogenesis, including synthesis of saturated C16 and C18 fatty acids (*Fasn* and *Elovl6*), and their conversion into MUFAs (*Scd1*). Many cancers, including those involving prostate and breast [24], [25], rely almost exclusively on *de novo* synthesis regardless of nutritional availability. The switch to *de novo* lipogenesis occurs early and is a prerequisite for efficient transformation. Novel effects of *OLR1* on lipid metabolism could account for much of its reported pro-oncogenic activity. For example, the expression level of *FASN* positively correlates with poor cancer prognosis [26], its genomic amplification is a common occurrence in some cancers [27], and its over-expression promotes transformation of epithelial cells [28]. Similarly, over-expression of *SCD1* has been observed in several types of cancers, including mammary cancer [29]; its upregulation is associated with transformation and its knock-down results in decreased cell proliferation, a loss of anchorage-independent growth and impaired apoptosis [30]. It is of note that compared to MCF10A cells, over-expression of *OLR1* in HCC1143 cells did not evoke the expected activation of lipogenesis genes. This may be explained by maximally increased basal expression of these genes in HCC1143 cells at baseline (Figure 3D).

As most of the genes upregulated in *OLR1*-TG HCC1143 cells are functionally pleiotropic, we evaluated the cumulative outcome of their upregulation on wound healing, adhesion and transendothelial migration assays (Figure 4). Notably, the migration of cells was seen to almost double the control value in cells with over-expression of *OLR1*, strongly suggesting a role for this molecule in breast cancer growth. On the other hand, presentation of *OLR1* on the surface of cancer cells did not seem to be essential for adhesion to activated endothelial cells or for transendothelial migration. The level of *OLR1* in endothelial cells, however, appears to be important, as addition of neutralizing *OLR1* antibody to the medium or inhibition of OLR1 transcription significantly impaired adhesion and transendothelial migration in non-transfected cancer cells. This is indicative of *OLR1* as a possible mechanism of cancer cell-endothelium interactions, as tumor cells are characterized by abundance of *OLR1* ligand phophatidylserine on the cellular membranes [31].

In summary, our data from multiple approaches in transgenic mice and human normal epithelial and cancer cell lines suggest that *OLR1* has several pro-oncogenic actions based on: a) activation of NF-κB signaling pathway resulting in inhibition of apoptosis and stimulation of proliferation; b) activation of de novo lipogenesis, and c) more efficient adhesion and transendothelial migration due to upregulation of *OLR1* in endothelium. We believe these data strongly suggest that *OLR1* may function as a link between obesity and susceptibility to breast cancer.

## Materials and Methods

### Animals

C57BL/6 mice were obtained from Jackson Laboratories. The homozygous *Olr1* KO mice were developed on C57BL/6 background as described previously [17]. The *Olr1* KO mice showed total absence of *Olr1* as determined by RT PCR and immunostaining, and the binding of oxLDL to the vascular intima was completely absent in these animals. All animals received humane care in compliance with the Public Health Service Policy on Humane Care and Use of Laboratory Animals published by the National Institutes of Health. The present studies were approved by UAMS Animal Care and Usage Committee approval number 2484, dated November 2007.

### Microarray Analysis

Total RNA of heart was extracted from WT mice and *Olr1* KO mice. Microarray analysis was performed by Affymetrix Mouse Genome GenChip 430 2.0 gene expression array (Affymetrix Inc. Santa Clara, CA) and analyzed using Affymetrix Microarray Analysis Suite (MAS) 5.0 to assess the quality of RNA and hybridization. A log base 2 transformation was applied to the data before the arrays were normalized. All values from each array were normalized to the 75th percentile value of the array, which was arbitrarily set at intensity minimum >100. For gene expression annotation, EASE (as described in http://apps1.niaid.nih.gov/David) analysis was performed on significant genes identified by one sample *t*-test. In addition, Gene Ontology (GO) terms http://www.geneontology.org) for biological processes and cellular component were identified as proposed by the GO Consortium. All microarray data is MIAME compliant and that the raw data has been deposited in a MIAME compliant database (ArrayExpress) as detailed on the MGED Society website http://www.mged.org/Workgroups/MIAME/miame.html (accession number E-MTAB-473).

### Reagents and cell lines

All reagents, unless stated otherwise, were purchased from Sigma (St. Louis, MO). Human breast cancer cell line HCC1143 was a kind gift of Dr. A. Basnakian (University of Arkansas for Medical Sciences, Little Rock AR). Mammalian expression vector (pCMV5-XL5) with human *OLR1* cDNA were obtained from Origene (Rockville, MD). Silencer Select Validated siRNA to OLR1 (s9843) was purchased from Invitrogen (Carlsbad, CA). Cells were cultured using standard RPMI 1640 growth medium supplemented with fetal bovine serum (10%) and ampicillin/streptomycin. High TBAR ox-LDL (90 nmoles MDA/mg Protein) was purchased from Biomedical Technologies Inc. (Stoughton, MA). Human IgG was purchased from Abcam (Cambridge, MA).

### Real-Time Quantitative PCR

Cells were transfected with either empty vector or *OLR1* expression vector. The efficiency of transfection was confirmed in parallel experiments with GFP carrying plasmid. Part of the transfected cultures (all in triplicates) was treated with oxLDL (40 µg/ml) for 24 hours before harvesting and RNA extraction. RT qPCR was performed using the Applied Biosystems 7900 real-time PCR system. qPCR specific primers were designed using Probe-Finder (http://www.roche-appliedscience.com) web-based software. All qPCR reactions were carried out in a final volume of 15 µl containing 1× of SYBR Green PCR Master Mix (Applied Biosystems, Carlsbad, CA), 300 nM of each gene specific primers, 100 ng cDNA, in sterile deionized water. The standard cycling condition was 50°C for 2 min, 90°C for 10 min, followed by 40 cycles of 95°C for 15 s and 62°C for 1 min. The results were analyzed using SDS 2.3 relative quantification manager software. The comparative threshold cycles values were normalized for GAPDH reference genes. qPCR was performed in triplicate to ensure quantitative accuracy.

### Transfection protocol

Cells were transfected with either empty vector or *OLR1* cDNA constructs (Origene, Rockville, MD) using lipofectamine 2000 (Invitrogen, Carlsbad, CA) in accordance with manufacturer's instructions with minor modifications. In preliminary experiments, we determined that higher transfection efficiency is achieved by applying a 2∶1 ratio of DNA to lipofectamine in relation to the proposed concentration of DNA recommended in the general protocol. Using these conditions, we routinely observed 70–80% transfection efficiency. Transfection of HUVECs with OLR1 Silencer (s9843) or scrambled siRNA (Invitrogen, Carlsbad, CA) was carried out using lipofectamine 2000 according to manufacturer's instructions. Cells we used in adhesion experiments 48 hours post-transfection and inhibition of OLR1 transcription was verified by quantitative RT PCR.

### Wound healing assay

Wound healing assay was performed to determine cell migration, according to the following protocol: Cells were cultured to confluence in 24-well plates, and two separate scratch wounds were made in every well using a sterile 200 µl pipette tip. Cells lifted in the process of scratching were gently removed by washing in PBS, then fresh growth medium was added. Pictures were taken at 10× magnification every 12 hours and the final picture after 36 hours of incubation was taken after loading the cells with calcein AM (Invitrogen, Carlsbad, CA).

### Adhesion assay

Human umbilical vein endothelial cells (HUVECs) were grown to confluence in 12-well plates and activated by exposure to oxLDL (50 µg/ml) for 4 hrs. Breast cancer cells HCC1143 were labeled with CellTracker Red CMTX (Invitrogen, Carlsbad, CA) according to manufacturer's instructions and added on to endothelial monolayer at concentration of 10^5^ cells per well. Plates were incubated for 1 hour in a CO_2_ incubator and then gently washed 3 times with growth medium to remove non-adherent cells. The number of cells attached to endothelium was counted in several fields of view in triplicate cultures using fluorescent microscope.

### Colorimetric transendothelial migration assay

The transmigration potential of HCC1143 cells was evaluated using QCM Tumor Cell Transendothelial Migration Assay (Millipore, Billerica, MA) according to manufacturer's instructions. Briefly, HUVECs were seeded on fibronectin-coated cell culture inserts at high density (pore size 8 µm), cultured until reaching 100% confluence and activated by exposure to 20 ng/ml TNFα.overnight. Breast cancer cells HCC1143 were added. (1×10^5^ per insert) to the monolayer and incubated in the cell incubator for 6 hours. Upon completion of the incubation period, growth medium and cells were gently swabbed from the interior of inserts The extent of transmigration was evaluated by measuring the amount of stain extracted from transmigrated cells on the outer surface of the membrane (absorbance at 570 nm) using a plate reader.
